# Designing the Hotspots Distribution by Anisotropic Growth

**DOI:** 10.3390/molecules26010187

**Published:** 2021-01-02

**Authors:** Tianshun Li, Renxian Gao, Xiaolong Zhang, Yongjun Zhang

**Affiliations:** 1 Key Laboratory of Functional Materials Physics and Chemistry, Ministry of Education, College of Physics, Jilin Normal University, Changchun 130103, China; lits0813@163.com; 2 Department of Physics, Xiamen University, Xiamen 361005, China; renxiangao@stu.xmu.edu.cn; 3 School of Material and Environmental Engineering, Hangzhou Dianzi University, Hangzhou 310012, China

**Keywords:** local surface plasmon resonance, SERS, hot spots, polarization-dependent effect

## Abstract

Changing the morphology of noble metal nanoparticles and polarization dependence of nanoparticles with different morphologies is an important part of further research on surface plasma enhancement. Therefore, we used the method based on Matlab simulation to provide a simple and effective method for preparing the morphologies of Au nanoparticles with different morphologies, and prepared the structure of Au nanoparticles with good uniformity and different morphologies by oblique angle deposition (OAD) technology. The change of the surface morphology of nanoparticles from spherical to square to diamond can be effectively controlled by changing the deposition angle. The finite difference time domain (FDTD) method was used to simulate the electromagnetic fields of Au nanoparticles with different morphologies to explore the polarization dependence of nanoparticles with different shapes, which was in good agreement with Raman spectrum.

## 1. Introduction

At a specific incident wavelength, the electric field near the surface of the noble metal can be amplified [1,2,3,4,5]. This phenomenon allows the molecular motion information around the nanoparticles to be amplified and detected, which is the main mechanism of surface-enhanced Raman scattering (SERS) [6,7,8,9]. The morphology of noble metal nanoparticles and the types of precious metals have an important influence on improving the local electric field strength of SERS [10,11,12,13].

To get higher enhancement factors (EF), many researchers are working on substrates with different morphologies. Many different shapes of substrates have been developed over the past years [14,15,16,17,18,19,20,21]. Li’s group [22] used experiments and theoretical studies to study the polarization effects of laser excitation on single nanowire (NW) plasma. The theoretical and experimental results showed the plasma-driven catalytic reaction could be controlled by changing the polarization of the excitation laser on a single anisotropic nanostructure, such as a single NW. Katyal et al. [23] used local time domain finite difference (FDTD) based theoretical models to study the localized surface plasmon resonance (LSPR) and localized field enhancement of ex vivo, dimeric, and multimolecular nanostructures. They found that spherical nanostructures exhibited great field enhancement in the DUV-UV region, while the anisotropically shaped nanostructures exhibited greater field enhancement and the peak wavelengths were tuned to the visible region. In fact, the preparation methods in these reports are too complex for the nanoparticles with different shapes, which wasted too much time in the preparation process. For the more important and various applications, the effect especially for anisotropic structures, polarization dependence plays an unneglected issue in SERS applications, not only in explanation of enhancement mechanism but in single-molecule detection. Therefore, it is very important to explore the polarization-dependent effects of these different shaped nanoparticle on SERS. Our group used the angle-dependent sputtering technique to prepare nanostructures with different shapes and microstructures on the PS nanosphere, and some interesting properties were observed15. We investigated the mechanism for the SERS polarization dependence of the Ag nanorods with different tilt angles. We demonstrated that the combination of two strong EM couplings area in the nanorod array was the root cause of the different polarization dependence observed for the nanorod arrays at different tilt angles. To understand and apply the hotspots more clearly, the ability to precisely determine the location of hotspots in an anisotropic metal nanostructure with a complicated configuration is very important. With the similar method, more different shapes of nanostructures were prepared in other researches to get good SERS substrates, such as nanostars [24,25,26,27], nanotube [19,28,29], nanoposts array [17,18,21], and so on.

Highly hexagonal closely packed polystyrene (PS, diameter 500 nm) spheres arrays were prepared on Si wafers using a self-assembly technique. Oblique angle deposition (OAD) was chosen for Au nanostructure preparation on the ordered PS arrays, and the Au nanostructures of the different shapes were created by changing the oblique angle. The polarization-related SERS observations were performed on these nanostructures, which is in good agreement with the time-domain finite difference method (FDTD) simulation, indicating we can control the polarization properties by a simple method.

## 2. Results

**Figure 1:**
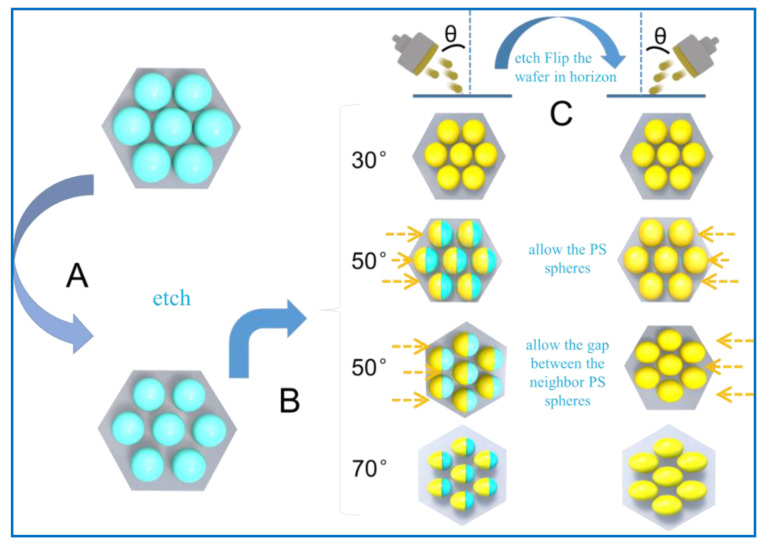
Schematic diagram for the preparation procedure of different shaped nanocaps array (**A**) monolayers on a Si wafer by a self-assembly method. (**B**) PS spheres separated by etching. (**C**) Au nanocaps with different shapes on the PS sphere arrays by depositing Au twice with different rotation angles.

As predicted by MATLAB simulation in Figure S1, the nanocaps of the different shapes were created on the etched PS beads array. Figure 1 shows the process to prepare the nanocaps of the different shapes based on a simple and efficient colloidal lithography method in a magnetron control sputtering system. First, the ordered polystyrene (PS) sphere arrays were prepared over a large area by a self-assembly technique. To get the different sizes for the nanocaps between the PS spheres, the PS monolayer was etched for different times by plasma etching. Finally, the OAD method was applied to deposit Au onto the etched PS array for the first time. The substrate was rotated by a certain angle at the original position and then the OAD deposition was applied again. As seen in the schematic diagram, the nanocaps of the different shapes were created when the different rotation angles were chosen.

Neighboring PS spheres were separated in different distances, with the diameter of PS sphere from 480 nm to 420 nm and the distance approximately from 20 nm to 100 nm. Nanoparticles of these shapes have been made by other groups such as Tian and his team. They used evaporating water on a two-dimensional Au nanoparticle array to control particle spacing and then experimentally and theoretically studied the relationship between SERS effect, SPR catalysis, and gap distance.

**Figure 2:**
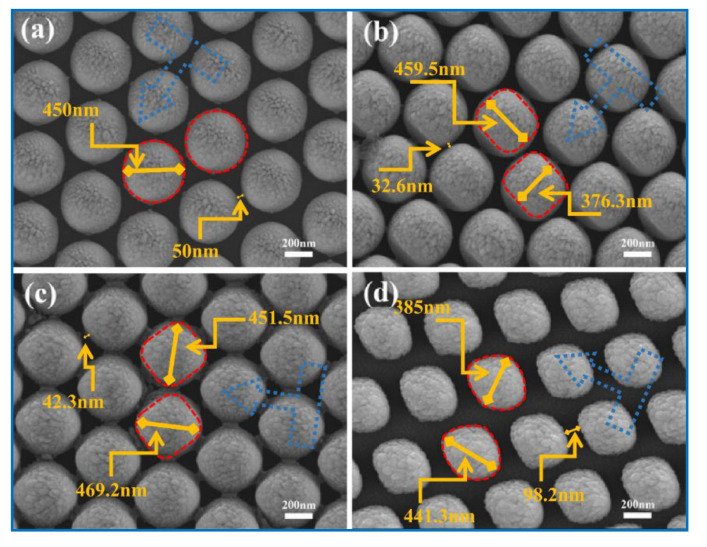
SEM images of nanocaps deposited on PS array after being etched for different times and with different OAD angles (**a**) 3 min, 30 degrees; (**b**) 3 min, 50 degrees; (**c**) 3 min, 50 degrees; and (**d**) 5 min, 70 degrees.

SEM images show the nanocaps of different shapes obtained for different OAD angles. When the OAD angle was 30 degrees, the shapes of the nanocaps were round, quite similar to those deposited with the OAD angle 0 degree, as shown in Figure S2. In addition, the sizes of the nanoparticles on top of the nanocaps were larger than those away from the tops of the nanocaps. When the OAD angle was 50 degrees, the nanocaps showed two kinds of shapes. When the deposition direction along the PS array was as indicated by the blue arrow in Figure 2b, the shadow effect by the neighbor PS sphere was significant. Therefore, the nanocaps showed the curved square shape in this condition. However, when the deposition direction was parallel to the gap between the neighbor PS spheres, the shapes of the nanocaps were greatly different from the shape shown in Figure 2b, because the shadow effect was significantly reduced and the nanocaps showed obvious preferred growth along the deposition direction as shown in 2c. The observations above show it is possible to create nanostructures with large aspect ratio when the deposition is performed with large OAD angle and reduced shadow effect. As the etching time increased to 5 min and the OAD angle increased to 70 degrees, the nanocaps showed significantly anisotropic shapes, like a walnut, as shown in Figure 2d. The size of the long axis was 441.3 nm, the short axis was 385 nm, and the distance between the neighbor units was 65.2 nm. This perfect walnut-shaped nanostructure array was observed over a large area, as shown in Figure S3. The TEM images show the shapes of the nanocaps in different deposition conditions.

In order to conduct the Raman test on the nanocap arrays with different morphologies, the excitation light with a wavelength of 632.8 nm was selected (according to the UV-vis, as shown in Figure S4). Characteristic vibrational modes, including ν(CC) ring-breathing modes (∼1070 and ~1575 cm^−1^), were observed in the Raman spectra of aqueous 4-MBA and SERS spectra of 4-MBA. In different gold nanocap array structures, the characteristic signals of 4-MBA occurred near 1588 cm^−1^, which was relatively significant. The optimal Raman peak strength at 1588 cm^−1^ was selected as the analysis basis. The shape of the nanocap changed from a circle to a rectangle to a diamond to a walnut, and the Raman strength gradually increased. This is because the tip appeared in the process of the change of the structure of the nanocap, which made the nanoparticles play a major role in the enhancement of the local electric field. At the same time, the polarization direction of the symmetry of the nanoparticles also changed with the change of the morphology of the nanoparticles. It is very useful to discuss the polarization dependence of nanometer cap with different planning directions.

**Figure 3:**
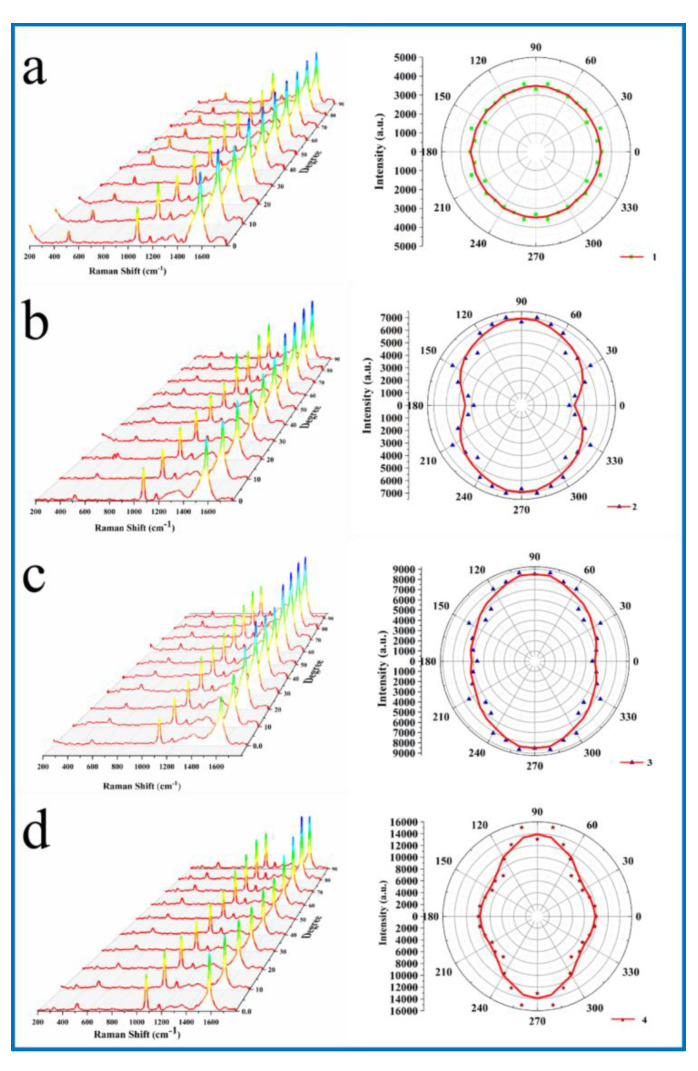
The left figure (**a**–**d**) is the spectrum diagram of SERS of 4-MBA adsorbed on samples of different shapes changing with the angle of excitation light, and the right figure is the polar coordinate diagram of corresponding SERS strength changing with the angle of incident excitation light.

In order to explore the polarization-dependent relationship of the structure of the nanocap array with different morphologies, the SERS intensity change of the nanocap array under differently polarized light was studied by changing the direction of the nanocap array under the laser. In order to display more clearly the SERS intensity change of the nanocap array under polarized light at different angles, we conducted a fitting analysis of the polarized light angle and the optimal Raman peak strength at 1588 cm^−1^, and the polar coordinates after fitting were shown in Figure 3 on the right side of a–d. By comparing the fitted polar coordinate graph with the SEM image, it is obvious that Raman intensities change with the polarization direction of the nanocap structure when the polarization direction of excitation light changes, that is, the fitted polar coordinate graph is consistent with the morphology of the corresponding nanocap.

**Figure 4:**
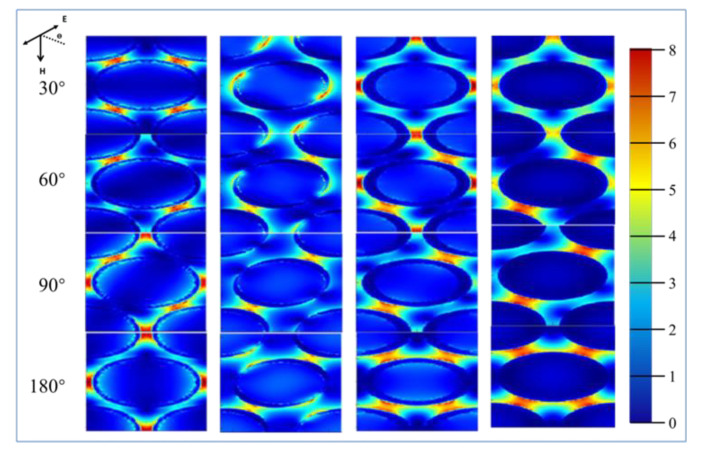
The electric field intensity under a polarized light source of FDTD simulations.

**Figure 5:**
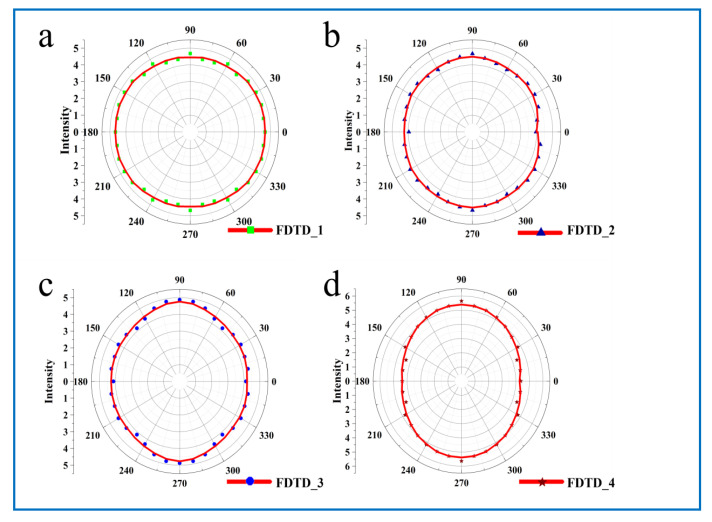
The (**a**–**d**) are the polarogram of electric field intensity of different polarized light incident at different angles obtained by finite difference time domain (FDTD) simulation of different shapes.

In order to prove the polarization dependence of nanoarrays with different morphologies when receiving polarized light from different directions, FDTD software was used to simulate the variation of local electric field intensity, as shown in Figure 4. The Ѳ is the rotation of the incident light in the x-y plane. Due to the large space between nanoparticles, the electric field coupling between nanoparticles is no longer the main basis for hotspot enhancement, but the electric field enhancement of individual nanoparticles is dominant in the nanoarray. Figure 4 shows the distribution of electric field intensity when the nanometer cap was a different shape, and when the incident light was polarized light from different angles. As can be seen, when the polarized light from different angles excited the circular nanocap structure, the electric field intensity was distributed around the nanoparticles. When the excitation angle of the line polarized light was changed, the position of the hot spot changes accordingly, due to the local plasmon resonance effect generated by the excitation of incident light, which enhanced the electric field around the nanoparticles. In order to clearly see the change of the intensity of the hot spot and polarization angle of polarized light, we fitted the mapping polar coordinate diagram of the intensity and polarization angle of the hot spot, as shown in Figure 5a. We found that the radar diagram simulated by FDTD and the graph of the fitting circle measured by experimental Raman fitted well with the experiment data.

When the deposition angle was 50°, the nanocap shape change was rectangular, when excited using a linear polarized light by changing the linear polarized incident angle, with similar behaviors as the spherical nano hat. The difference was that the distribution of hot spots tended to be more concentrated. When the polarization direction of the incident light was consistent with the “tip” area of the nanocap, the intensity of the hot spot was extremely strong. On the other hand, due to the short distance of the PS beads and the influence of the shadow effect, the Au nanoparticles deposited on the surface of the nanoparticles were more dispersed, leading to the enhancement effect of its own nanoparticles. By increasing the distance between the PS balls, the shadow effect produced was greatly reduced. This led to more areas where there were only a few Au nanoparticles before. The generated local plasma effect was stronger not because of the increase in the field strength between itself, but more the result of the mutual coupling between nano and nanoparticles. Under the influence of the tip effect, the field strength of the nano hat was further improved. In this square nanocap, there were mainly four significant enhancements of field intensity, which are caused by the effect at these four angles. Through the integration and analysis of FDTD results shown in Figure 5b,c, we find that the results were consistent with the laws measured in the experiment.

In order to obtain a sharper nanocap structure, we change the deposition angle to 70° to obtain a walnut-shaped nanocap structure. We also found a difference from the previous quadrangle nanocap when using linearly polarized light by changing the linearly polarized light’s incident angle. Originally, the enhancement effect of the field intensity at the tip of the nanoparticles was shifted due to the large distance between the nanoparticles, but this did not affect the enhancement effect of the field intensity of the nanoparticles. The integration of FDTD data shown in Figure 5d is consistent with the results of previous experimental tests.

## 3. Conclusions

In this work, a combination of etching time and variable angle sputtering was used to fabricate nanoparticles with different morphology. The basic laws of SERS enhancement of gold nanoparticles with different shapes and the polarization dependence of SERS under different incident light angles were studied. SEM image showed that the morphology of the nanoparticle changes from complete symmetry to axisymmetry as the sputtering tilt and etching time increase. The SERS spectra of nanoparticles with different symmetrical structures indicate strong polarization dependence. The results of SERS are confirmed by FDTD simulation and explained the reasons for the polarization dependence of gold nanoparticles with different morphology. Through the experimental and theoretical simulations of unified numerical data, we demonstrate that the appearance of strong EM coupling regions in nanoparticle arrays is caused by the coupling of the nanoparticles behind the tip and the sinking bottom. This study not only provides a simple preparation idea for the morphology of nanoparticles. Moreover, it further enhances the understanding of the existence of surface plasmon excitation in the symmetrical structure of nanoparticles, and has a broader application in the future of nano-optical refraction and nanosensors.

## 4. Materials and Methods

The monodisperse polystyrene colloid particles (PS, 500 nm) were bought from the Duke Scientific Corporation in Palo Alto, CA, USA. (10 wt.% aqueous solution). The chemicals, 4-Mercaptobenzoic acid (4-MBA) and ethanol, were bought from Sigma-Aldrich Co., Ltd., and the Au targets were bought from Beijing TIANQI Advanced Materials Co., Ltd. (HZTQ). Silicon (Si) wafers and ultrapure water (18.0 MΩ cm^−1^) were used.

The mixed solution (volume ratio NH_4_∙H_2_O:H_2_O_2_:H_2_O = 1: 2: 6) with Silicon wafers was kept in a beaker to boil for 5 min, and then the silicon wafers were washed by ultrapure water and ethanol three times, after which hydrophilic silicon wafers were obtained and stored in ultrapure water for future use. 100 uL PS aqueous solution was mixed with 100 μL ultrapure water, and a pipette was used to drop a suitable amount of the mixed solution onto the hydrophilic silicon wafer and then the wafer was slowly dipped into a container filled with ultrapure water. The polystyrene colloid particles formed a disordered monolayer on the water surface, which was driven by 2% sodium dodecyl sulfate solution to form a highly ordered monolayer on the surface. Finally, the ordered PS monolayer film was picked up by the clean silicon wafer, and the excess water was sucked up with the filter paper. After natural drying in the air, a highly ordered hexagonal densely stacked polystyrene template formed on the silicon wafer.

To fabricate the kinds of Au nanoparticles arrays based on the 2D PS templates, the 2D PS templates were etched by plasma etching for 1 min, 2 min, 3 min, 4 min, and 5 min, after which the neighboring PS spheres were separated by different distances. There was an included angle called OAD angle between the bottom template with the arranged balls and the deposited sediments. When different OAD angles are changed, one-time deposition is carried out, and then the initial nanocap structure after deposition is reversed 180°, and the final result is deposited again. Then, we used the OAD method twice to obtain the nanostructures of different shapes by rotating the substrate with an angle of 180 degrees.

For SERS observation, the gold nanoarray substrates were immersed in a 10–3 mol/L 4-MBA ethanol solution for 60 min, after which the samples were washed three times to clean the unabsorbed 4-MBA molecules. Finally, the sample was gently dried by N2 gas.

FDTD software was used to simulate the electromagnetic field distribution of the gold nanocaps array with different shapes. The geometric parameters of different structures were obtained from the experimental data. The periodic boundary conditions were used to simulate the region of the infinite two-dimensional nanoparticle array with different shapes placed on the x-y plane and the perfectly matched layer on the *z*-axis was used to absorb the reflected excitation light. The plane-polarized light rotating in the x-y plane was used as the excitation source, and the excitation wavelength was 632.8 nm. The refractive index of materials used in the simulation was all from the material database. In order to get the resolution of the electric field enhancement, the grid size was set to 2 nm.

SEM images were obtained on a JEOL-6500 F (JEOL LTD., Tokyo, Japan), using an acceleration voltage of 15.0 kV. Raman spectra were measured by a Renishaw Raman system, model 2000 confocal microscopy spectrometer (model 2000, Renishaw, London, UK) at a laser wavelength of 632.8 nm. The microscope was used to focus the laser beam onto a spot with a diameter of 1 μm with a 50× long-range objective. The equipment used a 180° backscattering geometry, and the time to collect the signal was set to 10 s. Ultraviolet-visible (UV–vis) spectra were obtained with a SHIMADTU ultraviolet spectrophotometer (UV-3600).

## Supplementary Materials

Figure S1: The MATLAB simulation of the different shape arrays, Figure S2: SEM images of nanocaps deposited on PS array with OAD angles of 0°, Figure S3: The large area of walnut-shaped nanostructure, Figure S4: U-V images of different nanocaps, Figure S5: The polar coordinate diagram of corresponding SERS strength changing with the angle of incident excitation light, when the wavelength was 518 cm^−1^, Figure S6. The polar coordinate diagram of the corresponding SERS strength changing with the angle of incident excitation light, when the wavelength was 1075 cm^−1^.
