# Supplementary material for: Designing the Hotspots Distribution by Anisotropic Growth
Source: Molecules. 2021 Jan 2;26(1):187. doi: 10.3390/molecules26010187 (PMC7795450; doi:10.3390/molecules26010187)
Supplement: Supplementary file 1 [file molecules-26-00187-s001.pdf]

Designing the hotspots distribution by anisotropic growth

Tianshun Li[1], Renxian Gao[2], Xiaolong Zhang[1*], Yongjun Zhang[3*]

[1] Key Laboratory of Functional Materials Physics and Chemistry, Ministry of Education, College of Physics,Jilin Normal University, Changchun 130103, China

[2] Department of Physics，Xiamen University，Xiamen 361005，China

[3] School of Material and Environmental Engineering, Hangzhou Dianzi University, Hangzhou 310012, China;yjzhang@hdu.edu.cn

* Correspondence: zhangxiaolong@jlnu.edu.cn (X. Zhang), yjzhang@hdu.edu.cn (Y. Zhang)

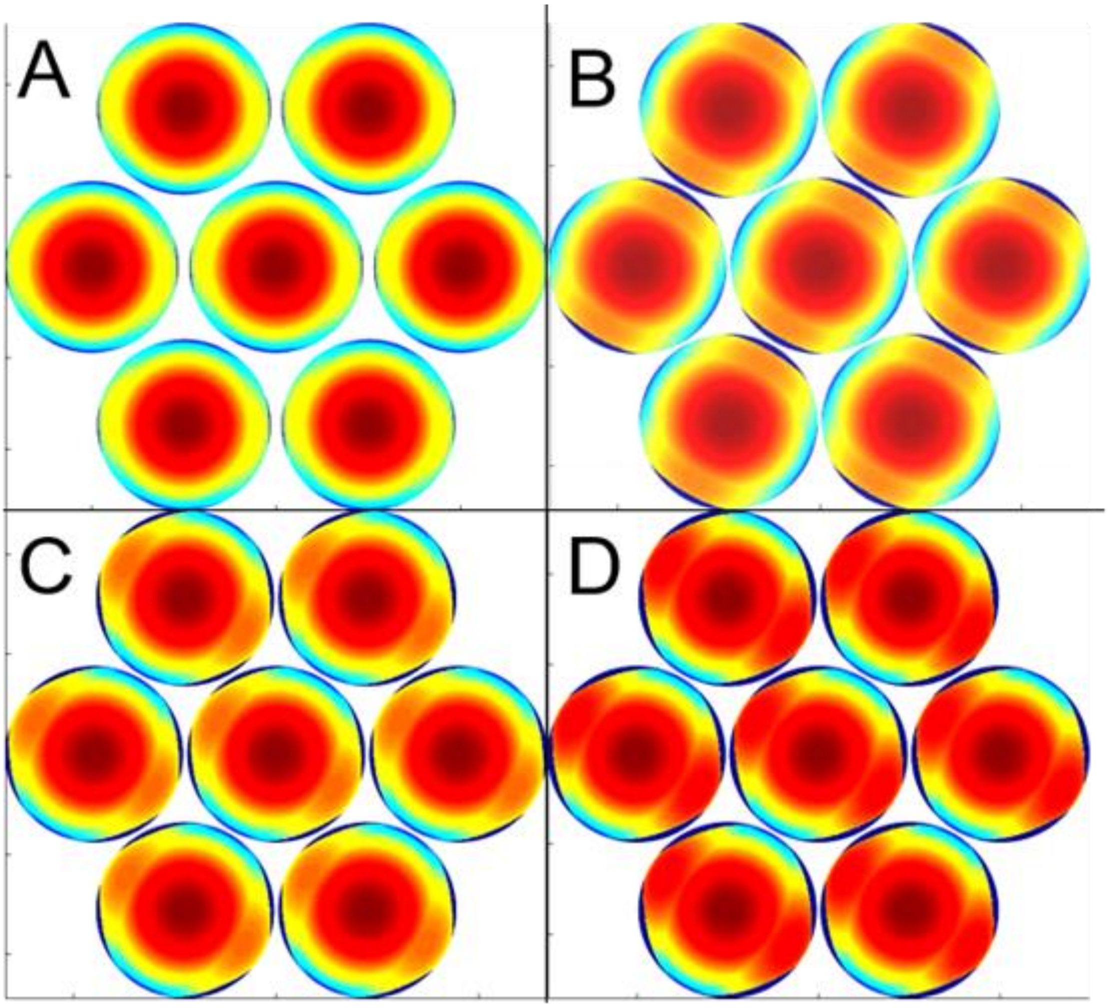

Figure S1. The MATLAB simulation of the different shape arrays.

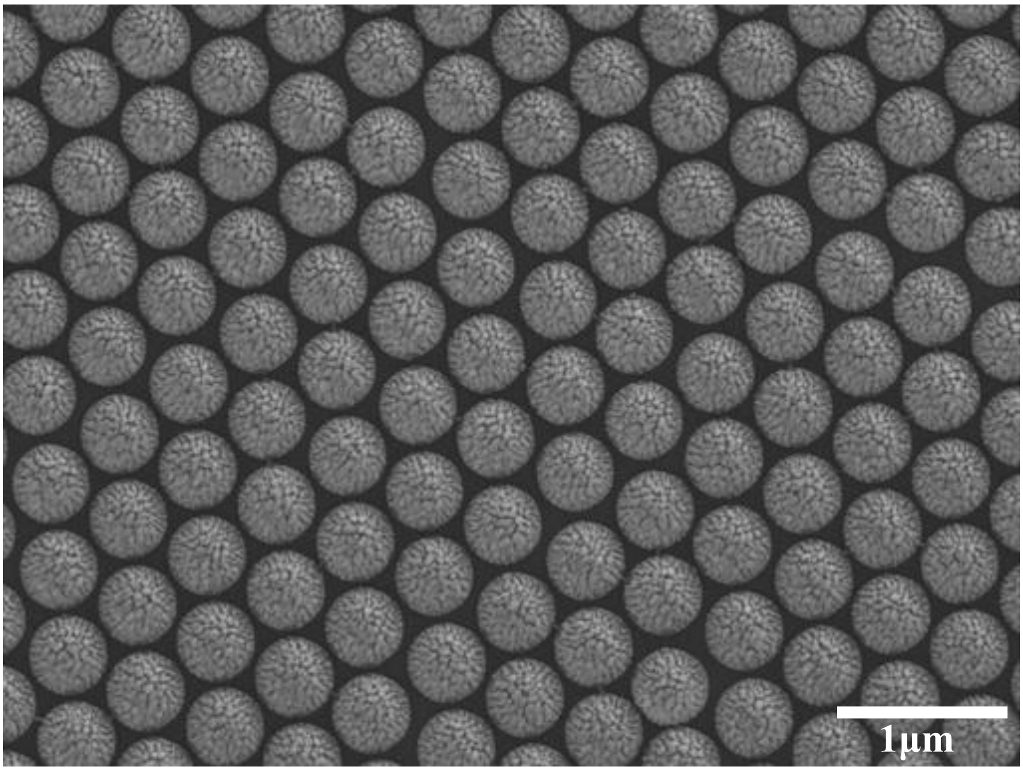

Figure S2. SEM images of nanocaps deposited on PS array with OAD angles of 0°

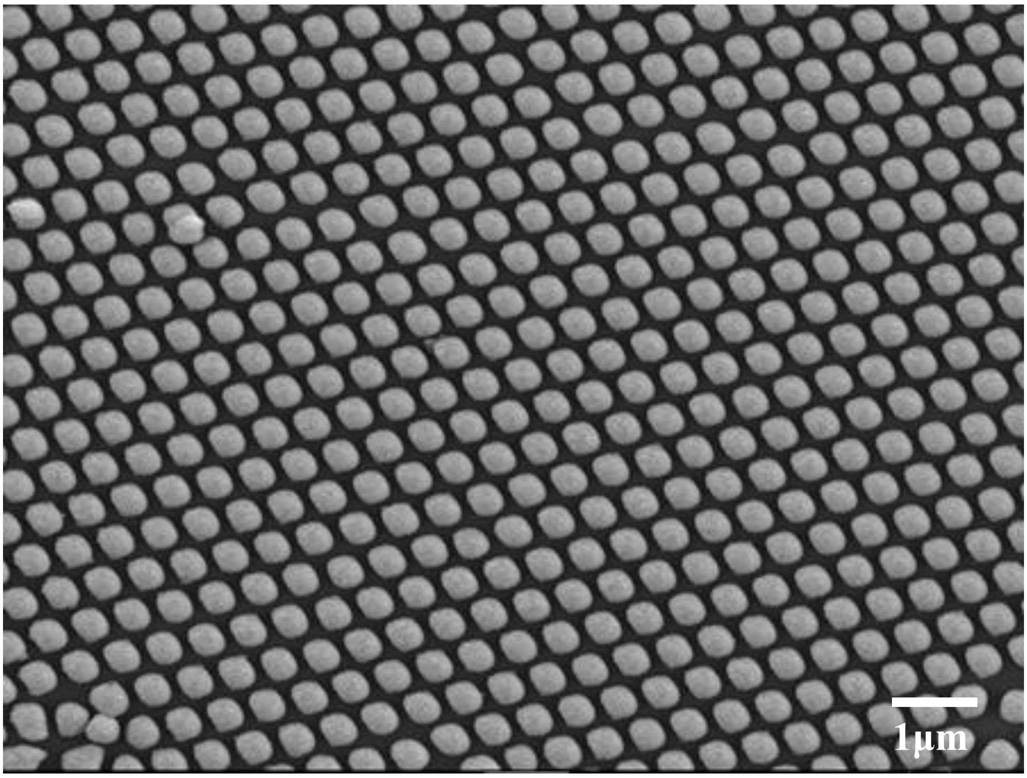

Figure S3. The large area of walnut shaped nanostructure.

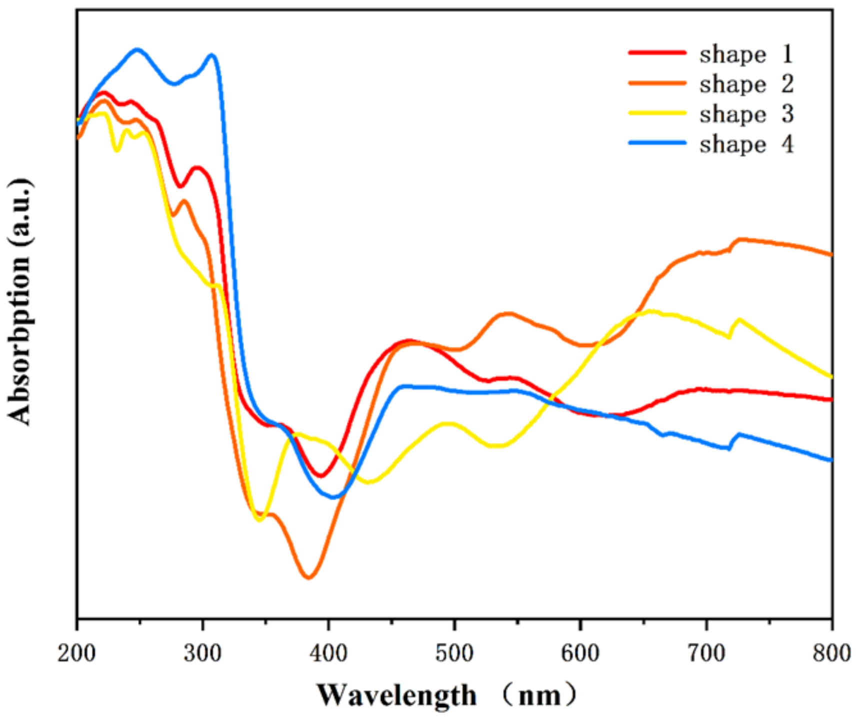

Figure S4. U-V images of different nanocaps.

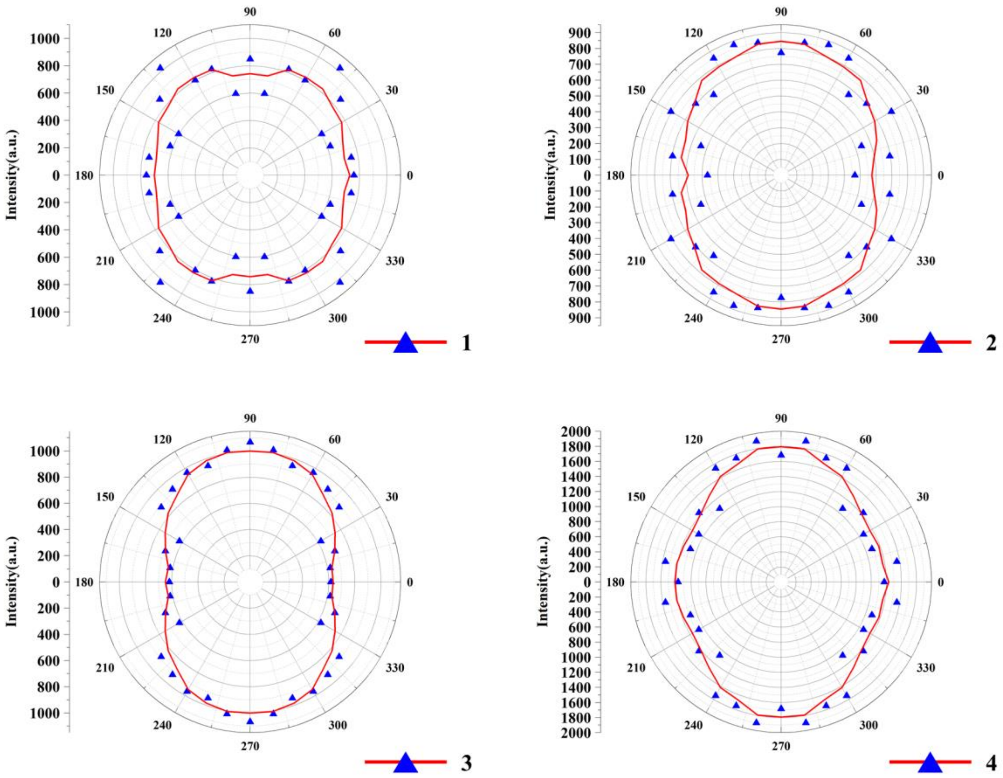

Figure S5. The polar coordinate diagram of corresponding SERS strength changing with the angle of incident excitation light. When the wavelength is the 518 cm^{-1}

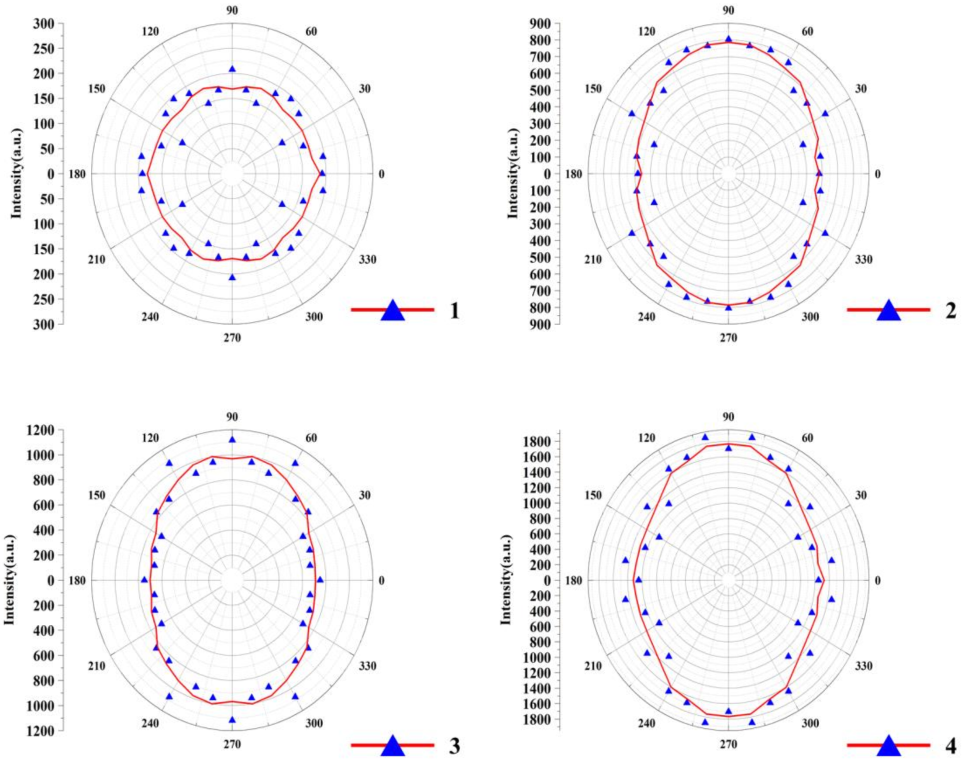

Figure S6. The polar coordinate diagram of corresponding SERS strength changing with the angle of incident excitation light. When the wavelength is the 1075 cm^{-1}
